# Increasing nitrogen limitation in the Bothnian Sea, potentially caused by inflow of phosphate-rich water from the Baltic Proper

**DOI:** 10.1007/s13280-015-0675-3

**Published:** 2015-05-20

**Authors:** Carl Rolff, Tina Elfwing

**Affiliations:** Stockholm University Baltic Sea Centre, 106 91 Stockholm, Sweden

**Keywords:** Bothnian Sea, Baltic, Nutrient limitation, Redfield, Eutrophication, Cyanobacteria

## Abstract

The study showed that the open water of the Bothnian Sea (BS) is likely to have shifted from altering nitrogen and phosphorous limitations of the spring bloom to more nitrogen-limited conditions during the last 20 years. This is affected by the by inflow of phosphate-rich and oxygen-depleted water from depths near the halocline in the northern Baltic Proper, where severe oxygen conditions currently cause extreme phosphate concentrations in the deep water. The change in relation between inorganic nitrogen and phosphorous in the BS occurs first in the deep water and then progresses to the surface water. The change can potentially cause increased production in the BS and more frequent cyanobacterial blooms. There does not appear to be any immediate concern in the short-term perspective for the state of the BS, but a progression of the processes may lead to a more eutrophic state of the BS.

**Electronic supplementary material:**

The online version of this article (doi:10.1007/s13280-015-0675-3) contains supplementary material, which is available to authorized users.

## Introduction

The purpose of this study was to investigate if the spring bloom in the Gulf of Bothnia (GoB, the northernmost part of the Baltic Sea) is potentially limited by nitrogen or phosphorous and if this regulation is changing. The high-level nitrogen treatment prescribed by EU directive (91/271/EEC) has not been required in the GoB because production in this water body has been considered generally phosphorous limited, whereas the Baltic Proper is nitrogen limited (Supplementary Material, Fig. 10.1007/s13280-015-0675-3). The export of nitrogen from the GoB to the nitrogen-sensitive Baltic Proper has also been estimated to be comparatively small (Savchuk 2005). Recurrent cyanobacterial blooms in the southern part of the GoB (e.g., Jaanus et al. 2011), however, suggest that the production regulation has changed. This study tests the hypothesis that the relation between inorganic nitrogen and phosphorous in winter nutrients of surface water has shifted toward more nitrogen-limited conditions, assuming the Redfield ratio to be optimal for phytoplankton growth.

**Fig. 1:**
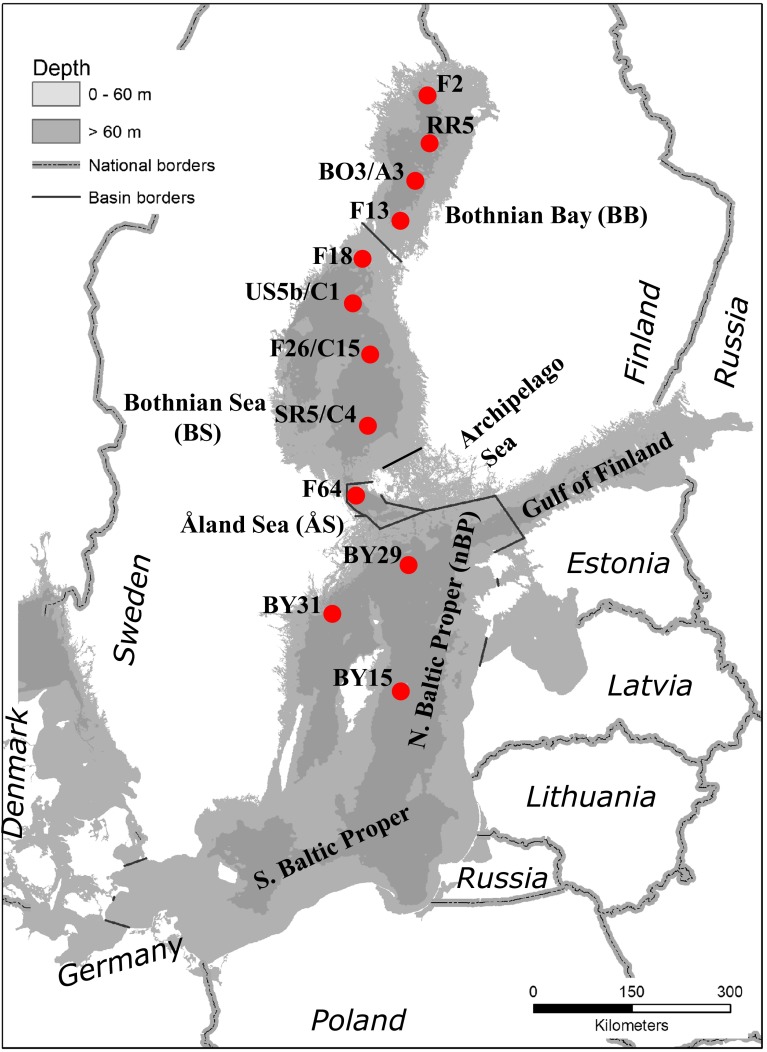
Map of the Baltic Sea indicating the Bothnian Bay (BB), Bothnian Sea (BS), Åland Sea (ÅS), Archipelago Sea, and the northern Baltic Proper (nBP), and monitoring stations supplying data to the study. The Gulf of Bothnia (GoB) consists of the BB, BS, ÅS, and the Archipelago Sea

The GoB is a large fjord-like, estuarine, nontidal water body. It is in the west, north and east enclosed by Sweden and Finland. The surface salinity decreases from 5 to 6 PSU in the south to 2 PSU in the north. It consists of two major basins separated by a sill: the northern Bothnian Bay (BB) and the southern Bothnian Sea (BS) (Fig. 1). The BB and BS, respectively, have volumes of 1481 and 4308 km^3^ and mean depths of 41 and 66 m (Leppäranta and Myrberg 2009). The residence time of water in the GoB is short and has for the BS recently been estimated to 4 years (Yi et al. 2013).

The BS connects in the south to the central basin of the Baltic Sea, the Baltic Proper which has exchange with the ocean through the Danish straits. The northern Baltic Proper (nBP) has surface salinities of 6–7 PSU. In the middle of this connection is the island of Åland. East of Åland is the shallow Archipelago Sea with an average depth of 19 m and a sill depth of 18 m (Leppäranta and Myrberg 2009). On the western side is the Åland Sea (ÅS) with a maximum depth of 300 m and connected to the BS through channels of 150-m depth (Leppäranta and Myrberg 2009) with the sill at about 90 m (Hietala et al. 2007). The information on the actual sill depth between the ÅS and the nBP differs between sources. Most sources give 45 m, whereas Leppäranta and Myrberg (2009) citing (Fonselius and Malm 1996) gives 70 m as the correct sill depth. The residence time of the Baltic Proper is on the time scale of 30 years (Stigebrandt and Gustafsson 2003).

The Baltic Proper has experienced symptoms of eutrophication since the 1960s caused by the external loading of nutrients from a variety of sources including sewage and agricultural runoff (Larsson et al. 1985; Conley et al. 2009). In combination with permanent stratification and the limited exchange with the ocean, severe problems occur with hypoxia and anoxia affecting extensive areas of sediment. Periodic anoxia occurs naturally in the deeper parts but major inflows of oxygenated and saline oceanic water intermittently replace the anoxic bottom water. If inflows are sufficiently frequent the deep water is replaced with oxygenated water but infrequent inflows may instead strengthen the halocline causing stagnation and hypoxia. During the period since the late 1970s, the frequency and the intensity of such inflows have decreased drastically (Matthäus et al. 2008). Since 1983, only three substantial inflows of water with high salinity have occurred (1993, 1997, and 2003) with the 1997 inflow being of moderate volume.

Low oxygen concentrations during stagnation periods cause denitrification of oxidized nitrogen and accumulation of ammonium and phosphate, which can reach 35 and 7 μmol L^−1^, respectively, in the deepest water. Phosphate from the deep water eventually causes elevated phosphate concentration in surface water, sometimes reaching 0.9 μmol L^−1^. High phosphate concentrations are a prerequisite for blooms of nitrogen-fixing cyanobacteria which occur in the summer (Bianchi et al. 2000; Kahru et al. 2007; Raateoja et al. 2011). These sometimes become very extensive and can in themselves be a nuisance for holidaymakers and tourists, but they also fix considerable amounts of nitrogen and thereby substantially contribute to eutrophication. Nitrogen fixation has been estimated to contribute around 300 000 tons of nitrogen annually (Larsson et al. 2001; Rolff et al. 2007) which is roughly a third of the total external nitrogen load on the Baltic Sea estimated by the Helsinki Commission (HELCOM 2009).

The phytoplankton production in the Baltic Proper has generally been found to be nitrogen limited (Granéli et al. 1990; Lignell et al. 2003; Tamminen and Andersen 2007; Danielsson et al. 2008; Walve and Larsson 2010). In particular, this applies to the spring bloom which is terminated when the inorganic nitrogen (DIN) supply is exhausted almost down to the halocline at 60–70-m depth, leaving some remaining phosphate (DIP) in the surface water above the developing thermocline. The nitrogen-fixing cyanobacterial summer blooms appear to be ultimately limited by the phosphate supply but also controlled by other factors such as temperature and the stability of the water mass (Kanoshina et al. 2003; Vahtera et al. 2005; Degerholm et al. 2006; Vahtera et al. 2010; Mohlin et al. 2012; Wasmund et al. 2012; Karlberg and Wulff 2013). The cyanobacteria also release considerable amounts of fixed nitrogen (Larsson et al. 2001; Rolff et al. 2007; Ploug et al. 2010). Limitation by both phosphorous and nitrogen can therefor occur which is difficult to study in the field or experimentally, since both nutrients occur at very low concentrations and are in rapid circulation. Both nitrogen and phosphorous may therefore potentially regulate production during summer, depending on the supply situation (Lignell et al. 2003; Vahtera et al. 2007; Walve and Larsson 2010; Raateoja et al. 2011).

There are very few studies of nutrient limitation in the GoB. The available information shows the BB to be phosphorous limited, but for the BS, the information is more complex (Fonselius 1978; Granéli et al. 1990; Andersson et al. 1996; Tamminen and Andersen 2007; Lundberg et al. 2009). The BS has traditionally been considered to be mainly phosphorous limited; however, nutrient-enrichment experiments found phosphorous limitation in coastal areas, but nitrogen limitation in the open sea (Andersson et al. 1996). There is also a gradient of increasing nitrogen limitation from north to south further reflecting the freshwater influence (e.g., Tamminen and Andersen 2007). Danielsson et al. (2008) found no time trends in the ratio of DIN:DIP in open sea surface water (0–20 m) during any season of the year for the time period, 1970–2001. Before 1991, they found winter DIN:DIP ratios both above and below 16 without any trend, but these ratios were consistently below 16 from 1991 to 2001, whereas Andersson et al. found a ratio of ~20 and ~10 in the northern and southern BS, respectively, in 1991. In a coastal study on the north-eastern side of the BS, phosphorous limitation was found in spring and autumn but nitrogen limitation in summer (Tamminen and Andersen 2007). The BS therefore appears to be a shifting zone from phosphorous-to-nitrogen limitation in the freshwater gradients both from coastal to open sea and from north to south, and the limiting nutrient can also shift between seasons.

## Materials and methods

Ideally, the nutrient limitation would be studied by the excess concentration of the nonlimiting nutrient in the production season. Regrettably, such data are not available for the BB and BS for recent years, and therefore the winter concentrations of DIN in relation to DIP were used to estimate the prerequisites for the spring bloom as discussed by, e.g., Granéli et al. (1990). A wide range of phytoplankton has remarkably constant composition of nitrogen relative phosphorous at the molar ratio 16:1. It is often referred to as the Redfield ratio (Redfield 1958), and it is useful to identify the limiting nutrient. Phytoplankton primary production is generally considered to be limited by nitrogen when molar DIN:DIP is below 16. Redfield ratios can, however, show variations between species and water bodies (Geider and La Roche 2002).

Using the DIN:DIP ratio in the water as an indicator of phytoplankton growth limitation has some disadvantages. Nutrient ratios are dimensionless and therefore give no quantitative information on nutrient availability. Particularly at low concentrations, ratios are also very sensitive to minute variations in the denominator (among those measurement errors), which can cause substantial change in the ratio. This can also give rise to statistical problems where ratios with low concentrations in the denominator may have strongly skewed distributions with sporadic extreme values causing high residual variation. In this study, we therefore use the difference between DIN and 16 times DIP measured in μmol L^−1^ as a study variable. The variable will here be called *Φ* (*Φ* = (NO_2_ + NO_3_ + NH_4_) – 16 × PO_4_). *Φ* is a difference and can be interpreted quantitatively as surplus or deficit of nitrogen and will have the same concentration unit as DIN and DIP. When *Φ* > 0, nitrogen occurs in excess and production is phosphorous limited; when *Φ* = 0, nutrients are balanced; and when *Φ* < 0, nitrogen availability limits the production. Accordingly a value of *Φ* = +1 μmol L^−1^ means excess of one micromole nitrogen, and *Φ* = −1 will mean a corresponding deficit. Surplus and deficit of one micromole phosphorous will, however, correspond to *Φ* = −16 and *Φ* = +16, respectively.

Changes in basinwide proportions of nutrients are generally small and slow and are therefore difficult to detect. To increase the sensitivity in the analysis and avoid potential systematic differences between laboratories, only data from the annual winter cruise (late November or early December) of the Swedish Meteorological and Hydrological Institute (SMHI) were used. Ideally January–February values should be used, but sampling is then generally prevented by ice. Being in a high arctic area, the changes in DIN and DIP this late in the year are small, and the obtained values of *Φ* were good estimates of winter conditions, which was verified by analyses of monitoring data from years with sampling also in January–February. Data are publicly available in the database SHARK at the website of SMHI with quality information and analytic method descriptions. The software Statistica 6.1 was used for regression analysis.

In this study, we have analyzed data from three stations in the northern Baltic Proper, one station in the Åland Sea, and four stations each in the BS and the BB (Table 1; Fig. 1). Three stations in the nBP were included for comparative reasons and to investigate the potential causality of identified changes in the GoB. These stations were sampled at the same cruises as the stations in the GoB. The station BY15 is located in the central Baltic Proper but is included in the study, since it is highly representative for large-scale processes in the Baltic Proper and will be treated as belonging to the nBP. Station BY29 is the most representative of water from the Baltic Proper entering the ÅS and continuing on to the BS. Data exist from 1969 but is sometimes intermittent and do not always contain complete estimates of DIN prior to a standardized national program initiated in 1993. The long-term development of *Φ* in surface water was therefore calculated for each basin for all available years and depths above 20 m using the 12 stations in four basin wide groups (nBP, ÅS, BS, and BB; Table 1). In the long-term data, there is some variation in the timing of the cruise from week 45 to 51 with a predominance of earlier cruises during the 1980s.

**Table 1:** Names of included stations from the Swedish national monitoring program. Geographic grouping in this study are indicated

Station name	Location	Grouping in this study
BY15	Central Baltic Proper	nBP
BY31	Northern Baltic Proper	nBP
BY29	Northern Baltic Proper	nBP
F64	Åland Sea	ÅS
SR5/C4	Southern Bothnian Sea	BS
F26/C15	Central Bothnian Sea	BS
US5b/C1	Northern Bothnian Sea	BS
F18	Northern Bothnian Sea	BS
F13	Southern Bothnian Bay	BB
BO3/A3	Southern Bothnian Bay	BB
RR5	Northern Bothnian Bay	BB
F2	Northern Bothnian Bay	BB

A more detailed analysis was made by individual stations for the years 1990–2012 (for some stations only from 1993) to study recent changes and the consistency of trends between stations. Analytic precision has increased compared to the period before 1990 particularly for nitrogen, and the timing of the cruise has been more standardized. For the more detailed analysis, only data obtained after week 47 were analyzed, and only the depths of 0, 5, 10, 15, and 20 m were included to give an unbiased value of the surface water. In a few instances, samples from 6 and 8 m replaced the 5 and 10-m levels because of initial differences in the sampling programs.

## Results

**Fig. 2:**
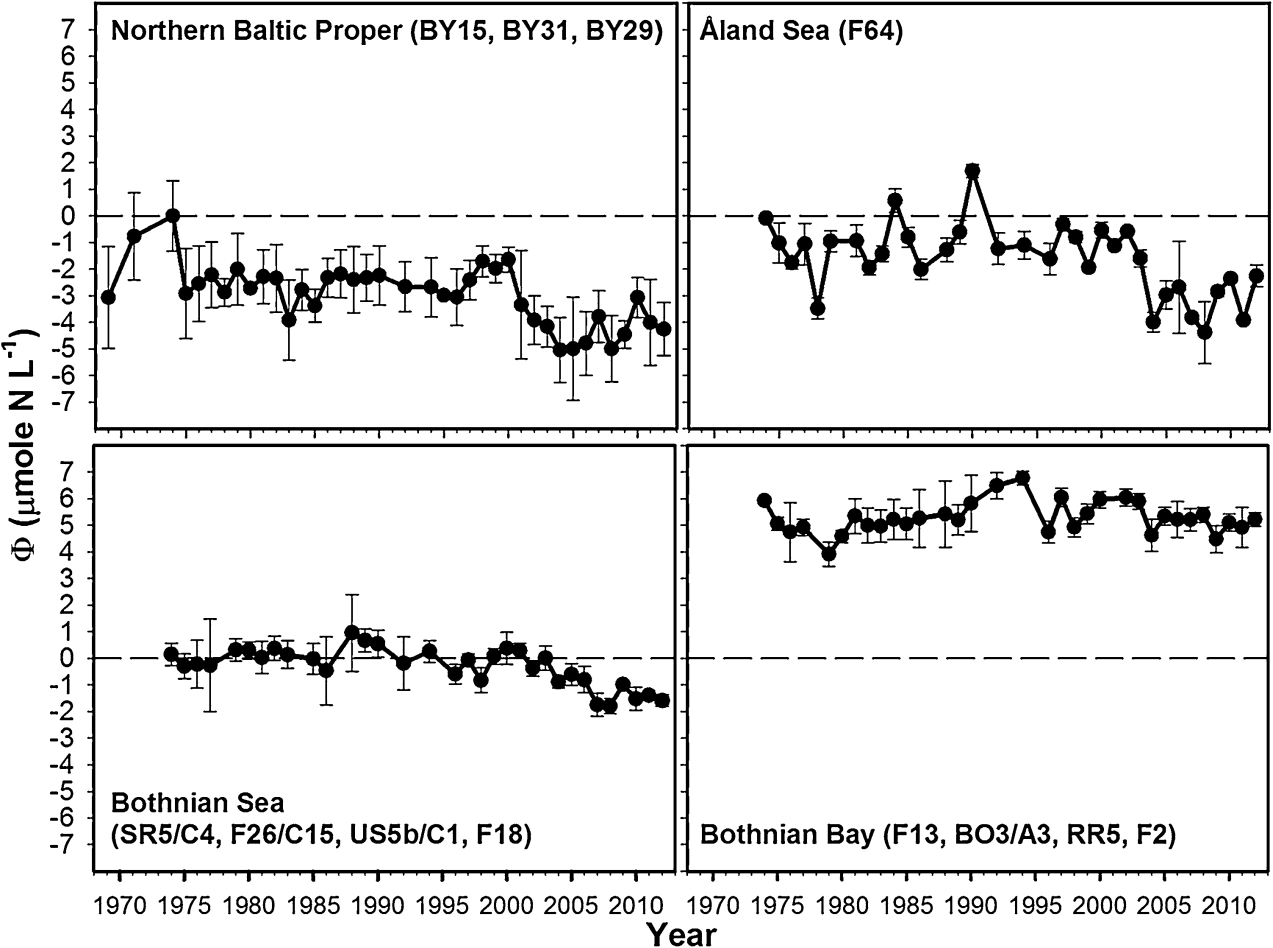
Long-term basin estimates of *Φ* (mean and SD) in winter surface water (November–December, 0–20 m) in the basins nBP, ÅS, BS, and BB using data from the winter cruise of the SMHI. Balanced nutrients (*Φ* = 0) indicated

A clear south-to-north gradient of *Φ* was found in the long-term analysis using all data available for surface water (0–20 m), going from strong nitrogen limitation in the nBP to strong phosphorous limitation in the BB (Fig. 2). The potential nutrient limitations differed distinctly between the basins BB, BS, and nBP, whereas the ÅS resembled the nBP (Fig. 2). In the nBP, winter values of *Φ* were strongly negative, indicating a strong nitrogen limitation of the spring bloom (lower quartile *Q*_25_ = −3.9 and upper quartile *Q*_75_ = −2.3 μmol N L^−1^). The ÅS showed slightly less negative values of *Φ* (*Q*_25_ = −2.4 and *Q*_75_ = −0.8 μmol N L^−1^). In the BS, the pattern was different and centered around nutrient balance of *Φ* = 0 (*Q*_25_ = −0.8 and *Q*_75_ = 0.26 μmol N L^−1^). The BB showed a clear surplus of nitrogen indicated by strongly positive values of *Φ* (*Q*_25_ = 4.9 and *Q*_75_ = 5.4 μmol N L^−1^). In the nBP, ÅS, and to some extent in the BS, *Φ* showed no clear temporal trend from 1969 to 2000 but an abrupt development toward stronger nitrogen limitation after 2000. In approximate terms, *Φ* in the nBP declined from −3 to −5 μmol N L^−1^ and in the ÅS from −1 to −4 μmol N L^−1^, whereas *Φ* in the BS changed from values around 0 to −1.5 μmol N L^−1^. The changes were the most pronounced in the nBP and ÅS with a weaker trend in the BS, whereas no long-term temporal trend was evident in the BB.

**Fig. 3:**
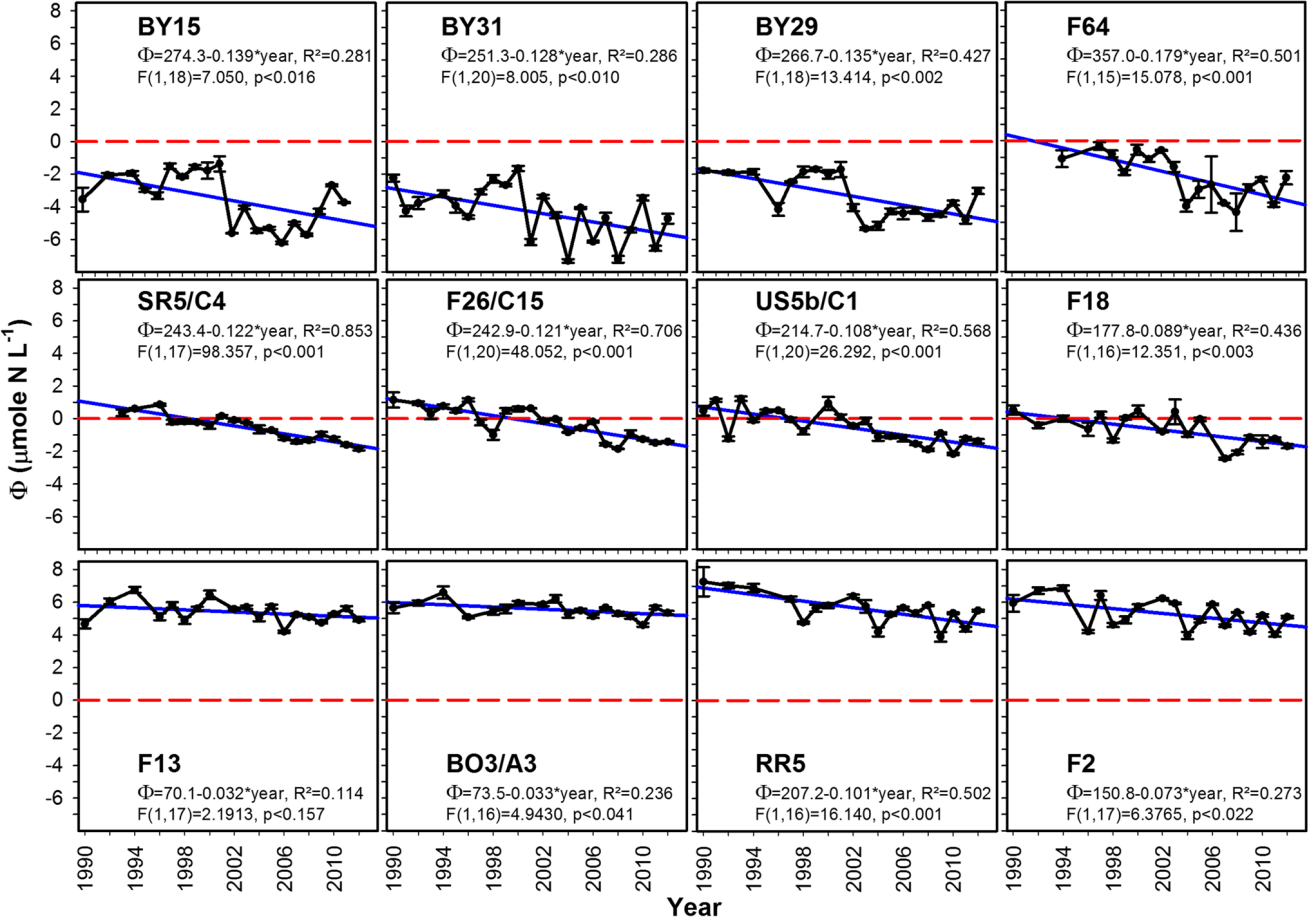
The development of *Φ* at individual stations (mean and SD) in winter surface water (0, 5, 10, 15, and 20 m) during winter from 1990 (F64 and SR5/C4 from 1993) to 2012. *Upper* nBP and ÅS, *middle* BS, *lower* BB. Balanced nutrients (*Φ* = 0) indicated

The general trends found at the basin scale could be recognized at the individual stations for the period 1990–2011 and were consistent for stations located in the same basin (Fig. 3). The change in *Φ* toward more pronounced nitrogen limitation around year 2000 could be identified at all stations in the nBP and ÅS. Even if regressions were significant, the regression explained variation was low (low *R*-square), because of a marked nonlinear shift around the year 2000. In the BS, the shift was gradual and showed a continuous decline without marked shift, and the linear time trends were highly significant (*p* < 0.003, statistics in Fig. 3). The intercepts (243, 243, 215, and 178 μmol N L^−1^) and slopes (−0.12, −0.12, −0.11, −0.09 μmol N L^−1^ yr^−1^) found in the BS were very similar for stations SR5/C4, F26/C15, US5b/C1, and F18, respectively. Slopes became less steep, and intercepts decreased going from south to north as did R-squares (0.85, 0.71, 0.57, and 0.44, for stations as above). In the BB, there were no strong temporal trends and low values of *R*-square. Yearly averages were used in the regressions to avoid unintentional weighting of years since there were sporadic occurrences of missing data. Results were, however, almost identical, if all data were used instead of yearly averages.

**Fig. 4:**
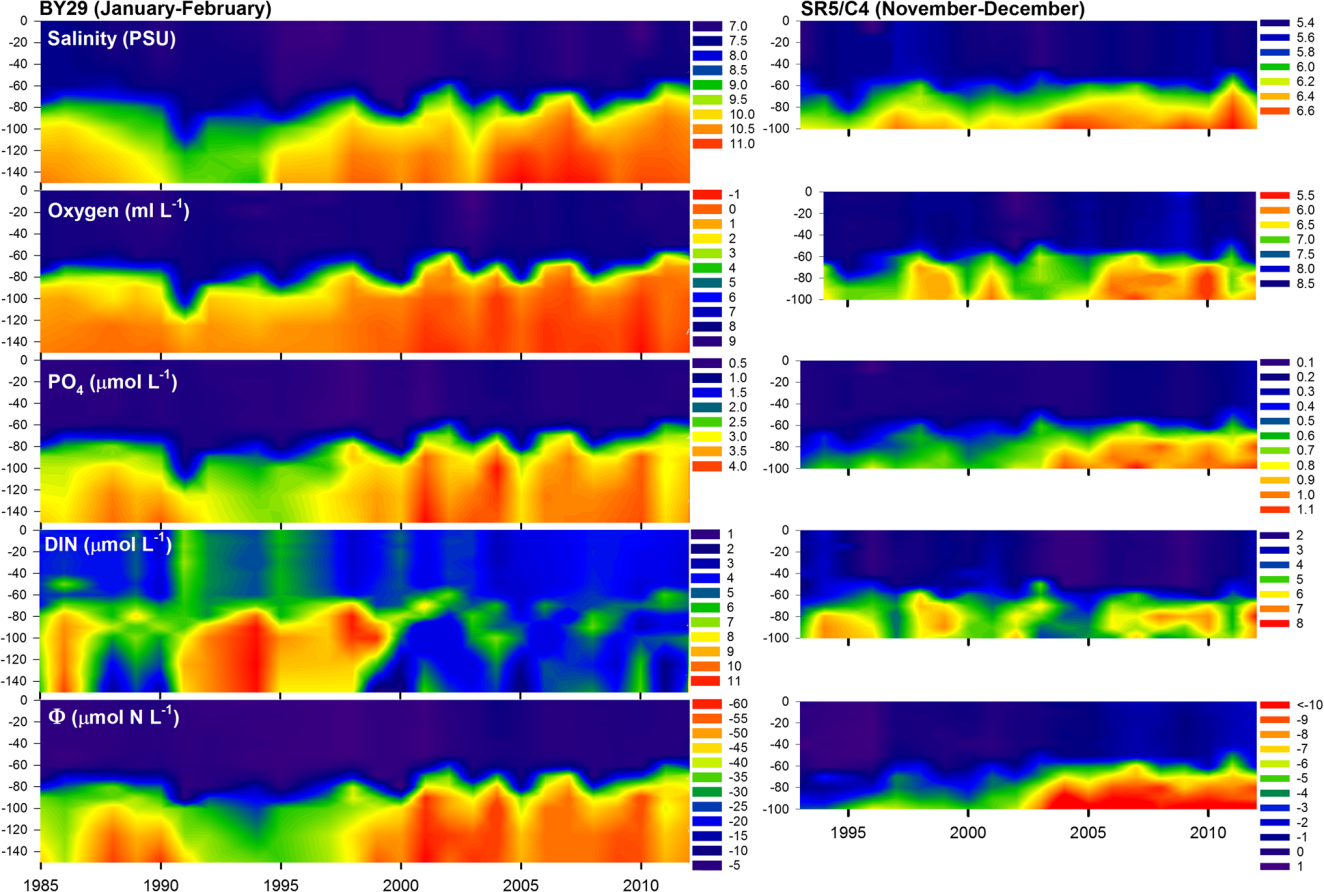
Salinity (PSU), oxygen concentration (ml L^−1^, oxygen required to oxidize H_2_S as negative oxygen concentration), phosphate concentration (μmol L^−1^), DIN (μmol L^−1^), and *Φ* (μmol N L^−1^) at stations BY29 (nBP) and SR5/C4 (BS). For station SR5/C4, November–December data were used, but for BY29, January–February data were used because of better data coverage and better representation of winter concentrations. Note that time and color scales differ between stations and that they have been cut to exclude extremes. The upper and lower levels of the color scales should therefore be interpreted as “or greater” and “or less” respectively

February values are generally considered as being the most representative for winter season’s inorganic nutrients. In this study, data obtained from the November to December cruise were used also for the nBP and ÅS with the exception of water column description as shown in Fig. 4. In the BB and BS, the winter has progressed considerably further during November–December than in the nBP. To ensure that there were no considerable differences in *Φ* values calculated from January to February, in relation to November–December values, a time series of monthly values from the years 1990 to 1999 were investigated. Such data were obtainable for stations BY31, US5b/C1, and station F9/A13 in the southern BB (not indicated in Fig. 1).

**Fig. 5:**
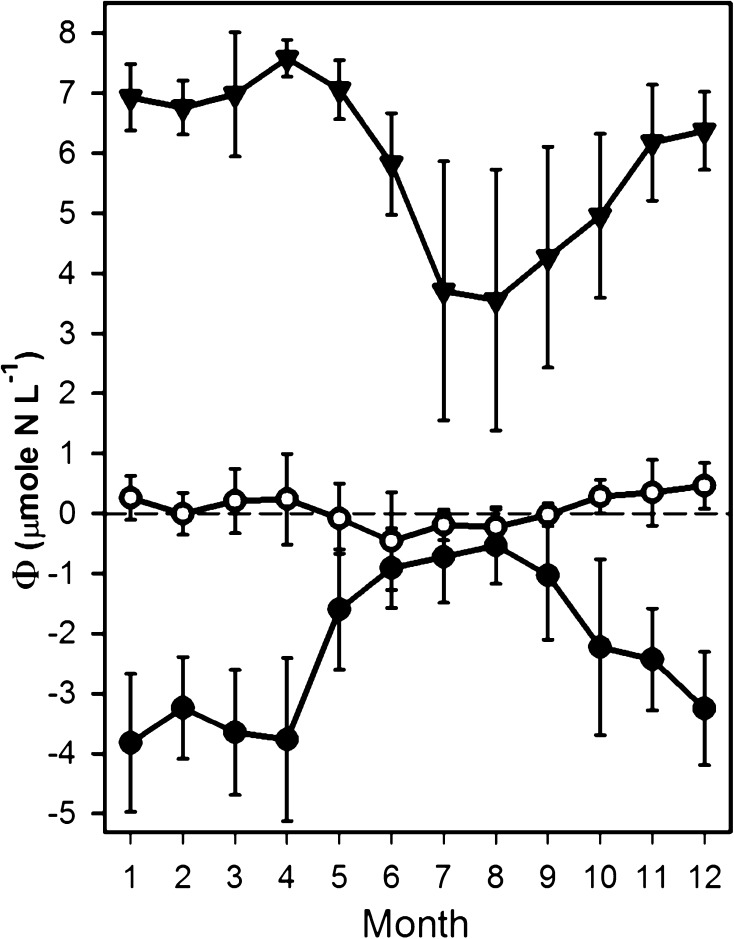
Monthly means of *Φ* (SD indicated) in surface water (0–20 m) at stations BY31 (nBP), SR5/C4 (BS), and F9/A13 (southern BB, not indicated in Fig. 1) for the years 1993–1999 when data where obtained for most years at a monthly basis

At station BY31, *Φ* values reached a minimum of ~−3.8 in January and a maximum in August of ~−0.5 (Fig. 5). The January *Φ* values were 0.6–1.4 μmol N L^−1^ lower than those during November–December. This is a substantial difference in relation to the difference between the minimum and the maximum. Potential nitrogen limitations of the spring bloom are thus underestimated in the nBP and ÅS when using November–December data compared with January–February. The difference between the nBP and the other basins is thus in reality greater than that indicated in this study. At the stations in the BS and BB, the differences in the values of *Φ* during January–February and November–December were small, and the latter are likely to be representative of winter conditions. In the BS, the November–December data represent a minor overestimate (~0.3 μmol N L^−1^) of *Φ* compared to February data, but only ~0.15 μmol N L^−1^ compared to January values. The February data were only available for 3 years in the time series.

## Discussion

### Causality behind the shift to nitrogen limitation in the Bothnian Sea

The area of seafloor in the Baltic Proper covered by anoxic water is now greater than that previously recorded (Hansson et al. 2013; Carstensen et al. 2014). Around 15 % of the seafloor is anoxic, and 28 % are hypoxic. The volume of hypoxic- and phosphate-rich water in the nBP has its upper limit at about 50–60-m depth, just above the halocline (Fig. 4). Cyclonic and estuarine circulations cause a south-going surface stream through the ÅS and a compensating north-going stream of above halocline water from the nBP that forms deep waters in the ÅS and BS. If the thresholds to the Åland Sea are indeed deeper than 45 m hypoxic, phosphate-rich water close to the halocline can periodically also enter the ÅS and contribute to the water overlaying sediments in the deeper parts of the BS. The corresponding salinity values of water found in the deepest parts of ÅS (F64) during the period (1990–2012) were found at between 50- and 60-m depths at BY29 in the nBP, showing passage of water from greater depths than 45 m in the nBP.

Oceanographic key variables in the nBP and the central BS showed a clearly parallel development (Fig. 4), with increasing deep water salinity, decreasing oxygen concentration, and increasing phosphate concentration. After the turn of the twenty-first century, the phosphate concentration increased dramatically below the halocline in the nBP because of anoxic or hypoxic conditions with a corresponding, but less consistent, increase in the surface water. Very high phosphate levels (up to ~3 μmol P L^−1^) were found close to the halocline at station BY29 in the nBP (Fig. 4). The trends in DIN are less clear, but the hypoxic conditions since the 1993 inflow caused nitrate to be denitrified in the deep water (e.g., Conley et al. 2009) followed by ammonium accumulation. The release of phosphate was, however, much more pronounced as can be seen from the progressively negative values of *Φ* at BY29 (Fig. 4).

**Fig. 6:**
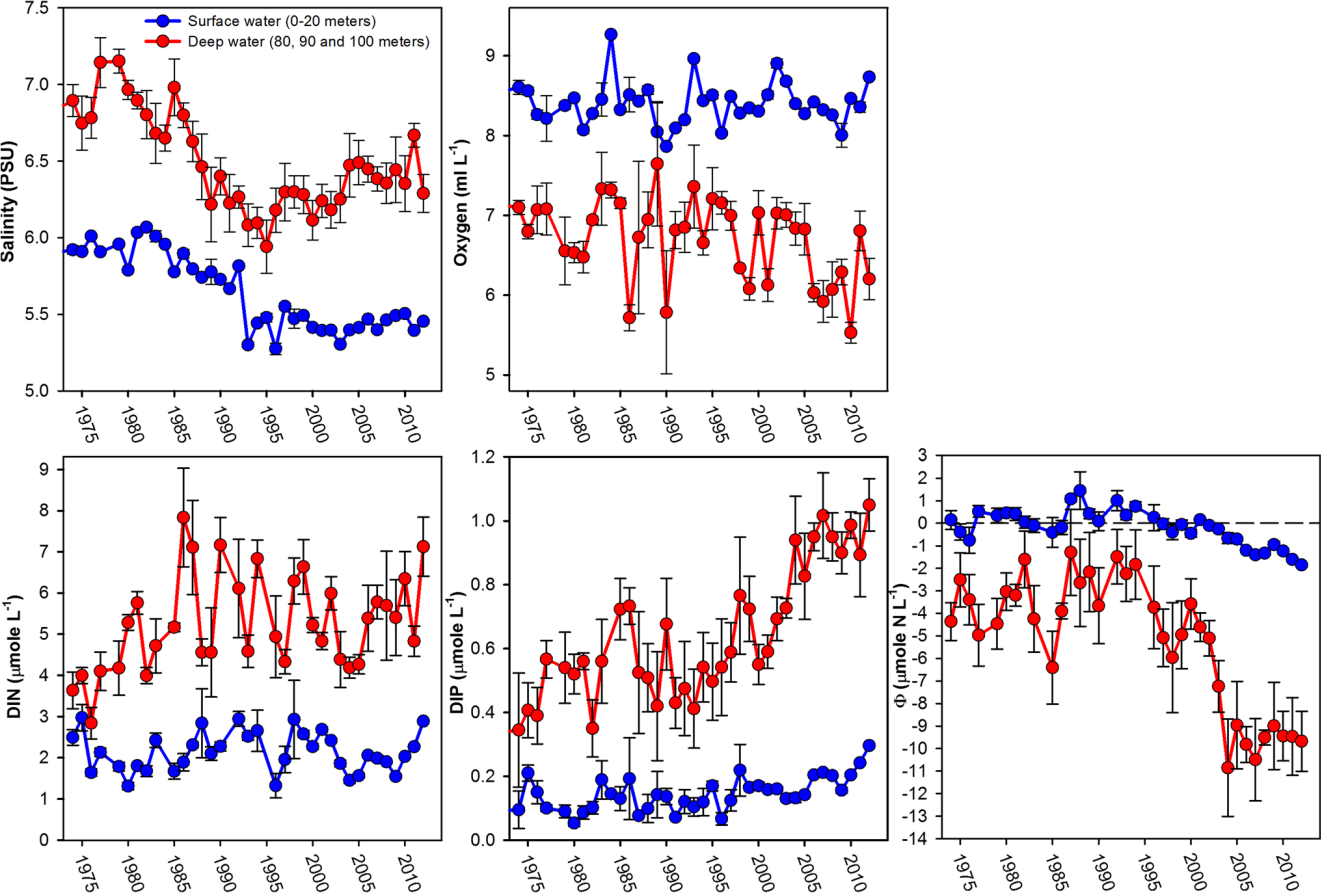
November–December salinity, oxygen, phosphate, DIN, and *Φ* values in surface water (0, 5, 10, 15, and 20 m) and deep water (80, 90, and 100 m) at station SR5/C4 (mean and SD). Changes in the deep water precede those of the surface water

From 1994 to 2012, there were no strong trends in external loads to the BS with uncertain estimates suggesting a somewhat greater relative reduction of phosphorous load in relation to nitrogen load on a weight basis (HELCOM 2013). The trends in the water column of the nBP, however, corresponded with damped equivalents in the BS (Fig. 4). The hypoxia and elevated concentrations of phosphate found at 50–60-m depth in the nBP are thus likely to be the main cause of the decreasing oxygen concentration and increasing phosphate concentration in the deep water of the BS (Fig. 4). The trend toward more nitrogen-limited conditions in the BS is likely to be driven by inflow of phosphate-rich water from the nBP, forming deep water in the BS since there is no consistent trend in DIN concentration in the water column (Fig. 6). For all the key variables, changes in the deep water precede the corresponding changes in surface water. Generally speaking, these are increasing deep water salinity, decreasing oxygen concentration below the halocline, increasing phosphate concentration, and variable trends in DIN. All trends were most pronounced below the halocline.

### Potential consequences in the Bothnian Sea

The BS has been considered as an area that can show both P and N limitations shifting between seasons and in a north-to-south and coastal-to-open sea gradient (Fonselius 1978; Tamminen and Andersen 2007). A reasonable assumption is that increased phosphorous load from the nBP would cause increased production. An extensive review found summertime chlorophyll-*a* in the BS to increase threefold from the late 1970s to the end of the 1990s (~1.1 to ~3.2 μg L^−1^), and then decrease again to ~2.4 μg L^−1^ during the period 2004–2006 (Fleming-Lehtinen et al. 2008). In a major review by the Helsinki Commission, chlorophyll-*a* was found to increase, and Secchi depth was shown to decrease for the period 1975–2007 (HELCOM 2009). National Swedish monitoring in the northern part of the open BS has shown a decreasing trend from around 2 μg L^−1^ in the early 1990s to values slightly above 1 μg L^−1^ during 2000–2004, and then a change back to values around 2 μg L^−1^ with considerable inter-annual variation and no clear time trends (Höglander et al. 2012). In a multivariate study of eutrophication indicators, coastal eutrophication was not found to be strong in the BS (Lundberg et al. 2009). For the GoB, the same study, however, found a north-to-south gradient of increasing eutrophication and an increasing trend in time.

As discussed above, there is no strong general trend for chlorophyll-*a* in the BS; most studies, however, suggest higher concentrations of chlorophyll-*a* during the last 10 years (Fleming-Lehtinen et al. 2008; HELCOM 2009; Lundberg et al. 2009; Höglander et al. 2012). Decreasing oxygen concentration in the deep water of the BS may suggest an increased production and sedimentation, causing oxygen consumption in the sediment. It is, however, unknown how much of the decrease in deep water oxygen concentration can be attributed to transport of oxygen-depleted deep water from the nBP and how much can be explained by local oxygen consumption in the sediment or deep water. In the deepest 30 m of water at station SR5, the oxygen concentration is slightly lower, and the phosphate concentration is slightly higher than those at 50-m depth in the nBP (BY29), suggesting also some local oxygen consumption and phosphate release in the BS.

The shift in nutrient limitation may lead to higher productivity in the BS. It is, however, essential to consider that the Redfield ratio is a general relation between nutrients in oceanic phytoplankton. The ratio has natural variations (Geider and La Roche 2002) and may thus have a value different from 16 in the BS. A study in the nBP found the molar N:P-ratio in seston to increase from values slightly below 16 in spring to 23 in late August (when diazotrophic cyanobacteria are frequent), and thereafter it decreased (Walve and Larsson 2010). The value 0 of *Φ* should therefore not be strictly interpreted as a distinct limit, and the movement toward more negative values of *Φ* is rather to be seen as a progressive shift toward increasing nitrogen limitation of the spring bloom. The values of *Φ* ~−2 found around year 2010 in the BS are, however, similar to those found around the turn of the century in the nBP, where the spring bloom is strongly nitrogen limited (Figs. 2, 3). The nitrogen-fixing cyanobacteria *Aphanizomenon* sp. has also been found to increase in the BS (Jaanus et al. 2011; Höglander et al. 2012) which generally is an indication of nitrogen-limited production conditions.

### Should the change cause immediate concern?

In the present situation, the results are not dramatic but should be closely monitored to evaluate if the winter pool of nutrients in the BS increases substantially, if chlorophyll-*a* will be found to increase and if the deep water oxygen levels continue to drop. During the 1990s, nutrients were measured monthly in the national Swedish marine monitoring program at station US5b/C1 in the northern BS. Analysis of these data showed that the DIN pool was almost exhausted down to approximately 50-m depth by the spring bloom which hereby consumes ~4 μmol N L^−1^ (56 μg N L^−1^). The volume of the BS down to 50-m depth is ~2770 km^3^ from which can be calculated an approximate consumption of 155 000 tons of DIN by the spring bloom. The annual load of total nitrogen to the BS by HELCOM is estimated to be ~50 000 tons N yr^−1^ of which roughly half the amount (~25 000 tons N yr^−1^) is considered as anthropogenic and the rest as natural background load from mainly forestland (HELCOM 2011). There is no basinwide estimate of inorganic load of nitrogen, but slightly less than a third is inorganic in the Swedish estimates of riverine load. The direct emissions from municipal wastewater treatment plants into the BS is estimated to be ~2100 tons N yr^−1^ (HELCOM 2011). In relation to the nitrogen consumed by the spring bloom, it is a comparatively small amount. However, nitrogen in emissions from MWWT generally consists almost exclusively of inorganic nitrogen. The MWWTs can therefore not be considered a major source of total nitrogen but may locally be an important source to inorganic load.

The increasing amount of phosphate is likely to cause some increase in spring bloom production in the BS. If increased sedimentation does not cause any significant decline in deep-water oxygen concentration and it remains sufficiently high to stabilize phosphate by oxidized iron, then the change can be seen as a mechanism by which the Baltic Proper can export excess phosphate to the BS, which is considered as only mildly eutrophic. The decreasing oxygen levels of the BS would in this perspective be caused mainly by transport of near halocline water from the nBP and not by increased local oxygen consumption. Increased production and nitrogen limitation would further decrease the relatively small transport of nitrogen to the nitrogen sensitive Baltic Proper. This net transport has been estimated to be 31 000 tons N yr^−1^ (Savchuk 2005) which is ~5 % of the external load on the Baltic Proper of ~350 000 tons N yr^−1^ (excluding the Gulfs of Finland and Riga) (HELCOM 2012) plus the internal load by nitrogen fixation of ~300 000 tons N yr^−1^ (Larsson et al. 2001; Rolff et al. 2007). If on the contrary local oxygen consumption in the deep water of the BS is significant, then the BS may come to suffer from similar but less-severe problems as the Baltic Proper. The short residence time of the water, weaker halocline, more efficient winter circulation, and lower production of the BS compared with the Baltic Proper are, however, likely to mitigate severe oxygen conditions in the deep water (Raateoja 2013). It is, however, not well known how the release of phosphate from the sediment is affected by moderate decreases in oxygen concentration in the deep water.

The clear similarity in the temporal development of all variables between station SR5 and BY29 strongly suggests that the nBP drives the observed changes. To what extent internal processes contribute to the development cannot be evaluated presently. A situation may be reached where also the nitrogen loads on the BS must be considered if the development toward a nitrogen limited production continues and potentially is reinforced by internal processes.

## Conclusions

The study showed a shift toward more nitrogen-limited conditions for the spring bloom in the BS during the last 20 years. The change in DIN relative DIP occurs first in the deep water and then progresses to the surface water suggesting that it is caused by inflow of phosphate-rich and oxygen-depleted water from depths near the halocline in the nBP and not by changes in external input. The change can potentially cause some increased production in the BS and potentially increase cyanobacterial blooms. There does not appear to be any immediate concern in the short-term perspective for the state of the BS, but a progression of the processes could lead to a more eutrophic state of the BS.

## Electronic supplementary material

Supplementary material 1 (PDF 237 kb)
