# Supplementary material for: Increasing nitrogen limitation in the Bothnian Sea, potentially caused by inflow of phosphate-rich water from the Baltic Proper
Source: Ambio. 2015 May 20;44(7):601–11. doi: 10.1007/s13280-015-0675-3 (PMC4591228; doi:10.1007/s13280-015-0675-3)
Supplement: Supplementary file 1 — Supplementary material 1 (PDF 237 kb) [file 13280_2015_675_MOESM1_ESM.pdf]

Electronic Supplement Material

Increasing nitrogen limitation in the Bothnian Sea, potentially caused by inflow of phosphate-rich water from the Baltic Proper

Carl Rolff, Tina Elfwing

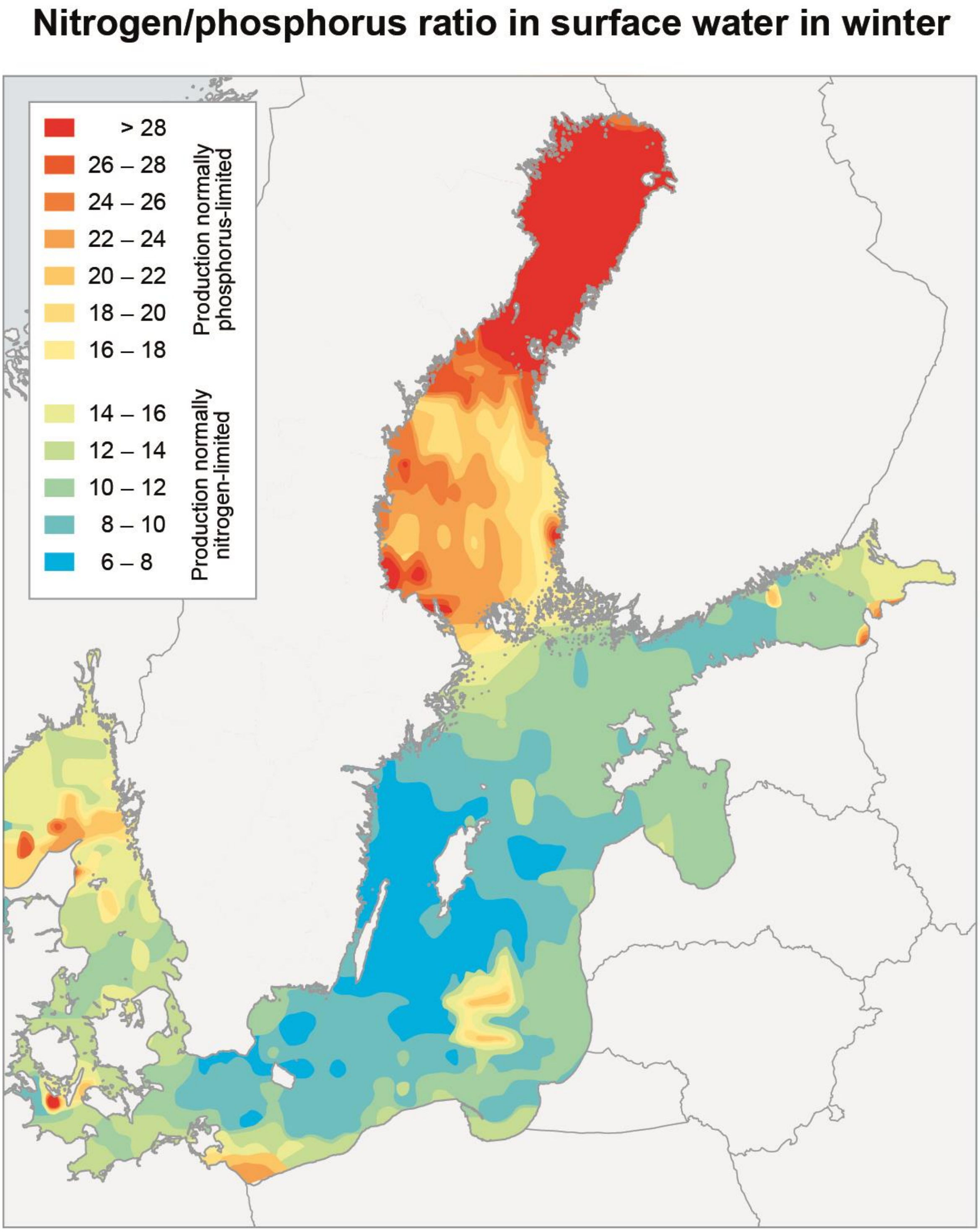

Figure S1

Molar nitrogen/phosphorous ratio in winter surface water in the years 1995-2002. Reprinted with permission from: Claes Bernes (2005) *Change beneath the surface : an in-depth look at Sweden's marine environment*, Swedish Environmental Protection Agency, Stockholm. Data from the Department of Systems Ecology, Stockholm University.
